# Survey of Dermatophytes in Stray Cats with and without Skin Lesions in Northern Italy

**DOI:** 10.1155/2014/565470

**Published:** 2014-05-13

**Authors:** Daniela Proverbio, Roberta Perego, Eva Spada, Giada Bagnagatti de Giorgi, Alessandra Della Pepa, Elisabetta Ferro

**Affiliations:** Dipartimento di Scienze Veterinarie per la Salute, la Produzione Animale e la Sicurezza Alimentare, Università degli Studi di Milano, Via G. Celoria 10, 20133 Milano, Italy

## Abstract

The aim of this study was to determine the prevalence of dermatophytes in stray cats with and without clinical lesions from different colonies in rural and urban areas of Milan and surroundings in northern Italy. Stray cats (273) were caught during a trap-neuter-release (TNR) program conducted in different colonies of northern Italy in both rural and urban areas. Each cat was examined in dark environment with a Wood's lamp prior to sample collection. Hair or scales exhibiting typical fluorescence were removed with a pair of sterile hemostats and cultured. The hair of all cats was then sampled by Mackenzie modified brush technique regardless of the presence or absence of skin lesions attributable to dermatophytosis. All the hair samples were subjected to fungal culture. 15 cats were positive (5.5%). *Microsporum canis* was the most common dermatophyte isolated (13/15). The only other isolated dermatophyte was *Trichophyton mentagrophytes* (2/15). Our estimated prevalence of dermatophytes in stray cats was much lower than other Italian studies on the same population.

## 1. Introduction

Various fungal organisms, as dermatophytes or saprophytes fungi, are frequently found on the feline hair coat [1, 2]. The most diffuse zoophilic dermatophytes that are primarily animal pathogens belong to the* Microsporum* and* Trichophyton* genera and some species of them may cause dermatophytosis in humans [1, 3].

The prevalence of dermatophytes in cats has been reported with much variability, depending on geographical location, season of sampling, and clinical and living conditions [4].

There are few data regarding the prevalence of* Microsporum canis* in stray cats. In asymptomatic or random screening stray cats the world prevalence of dermatophytes varies from 5% to 50% [5–10]. In Italy, dermatophytes* were* isolated from 27% to 50% of stray cats regardless of the presence of clinical sings [7, 9].

In most worldwide and Italian studies,* M. canis* is the most isolated dermatophyte in stray cats and its prevalence varies from 0% to 47.4% [5–10] while* Trichophyton mentagrophytes* is rarely isolated and its prevalence varies from 0% to 11.9% [5–10].

The knowledge of health status of free-roaming stray cats is important in the assessment of animal welfare and to obtain information about pathogens and diseases located in each environmental region.

The aim of this study was to improve the information regarding the prevalence of dermatophytes in stray cats. We have evaluated a large sample of stray cats in northern Italy, since this area has not previously been examined.

## 2. Material and Methods

### 2.1. Feline Population

Hair samples were collected from 273 stray cats with and without clinical signs living in northern Italy. The cats were caught by volunteers between April 2008 and February 2010 during a trap-neuter-release (TNR) program approved by the local authority of the city council, conducted as described previously [11]. The colonies were located both in rural and urban areas of Milan and surroundings in areas adjacent to human structures such as schools, hospitals, farms, and homes.

Cats were anaesthetized and, before surgery, were evaluated, by clinical examination, for general health and presence or absence of ectoparasites. Unhealthy cats were defined as cats with the presence of one or more of the following clinical abnormalities: lymph node enlargement, pale mucous membranes, stomatitis, or signs of ocular and respiratory infections.

### 2.2. Sample Collection

After anaesthesia each cat was examined in a dark environment with the Wood's lamp for several minutes prior to sample collection. Hair or scales exhibiting typical fluorescence were removed with a pair of sterile hemostats and cultured. Then the hair coat of all 273 cats was brushed with a sterile toothbrush using the modified Mackenzie collection method [1, 12].

To evaluate seasonal trends in dermatophyte infection, the samples from each group were categorized according to the sampling period as either warm season samples (collected from April through September) or cold season samples (collected from October through March).

### 2.3. Fungal Culture

All the hair samples were inoculated by gently imprinting the toothbrush onto the surface of 9 cm Petri dishes containing Sabouraud dextrose agar (added with chloramphenicol 0.5% and actidione 0.4%). The Petri dishes were incubated upside down in an oven (MICRA, I.S.Co., Zetalab S.R.L., Italy) in the dark at a constant temperature of 25°C and examined daily for three weeks. After three weeks the colonies in the medium were macroscopically and microscopically examined and identified to species level as dermatophytes.

### 2.4. Statistical Analysis

The Fisher's exact test was used to evaluate differences in the prevalence of the different species of isolated dermatophytes. Epidemiological data from cats were analysed by Chi-square test, in order to identify significant differences between the observed dermatophytes prevalence according to age, gender, breed, hair coat length, skin lesions, habitat, and season. The software used was MedCalc (Version 11.6.1.0, Mariakerke, Belgium) and *P* value < 0.05 was considered significant.

## 3. Results

Age, gender, breed, hair coat length, and habitat (urban or rural areas) were recorded for all cats (Table 1). Age was estimated by dentition and animals were classified as young (<1 year of age) and adult (>1 years of age) [13].

The prevalence of dermatophytes on the hair coat of overall stray cats of our study in northern Italy was 5.5% (15/273). Only two dermatophytes were cultured and both were zoophilic:* Microsporum canis *(86.7%—13/15) and* Trichophyton mentagrophytes* (13.3%—2/15) (Table 1). Sex, age, hair coat length, season of sampling, and geographical habitat did not show a significant association with dermatophytes prevalence. There were no positive cultures among cats identified as positive (20/273) under Wood's lamp. The ectoparasites found in cats were flea (9.16%—25/237) and tick (1.46%—4/237).

## 4. Discussion

The prevalence of dermatophytes in our study, on stray cats in northern Italy, was 5.5% which is lower compared with the prevalence found in other studies conducted on stray or feral population (35.2% in New Zealand [5], 25.6% in feline colonies of Iran [6], and 29.4% in Portugal [10]), but similar to the prevalence of 5% found in the UK [8].

When considering only the Italian stray cats studies, the prevalence of dermatophytes is much higher compared with our result. A study conducted in different parts of Siena (central Italy) found a prevalence of 50% in asymptomatic stray cats [7] and a stray cat study conducted in the north east of Italy detected a prevalence of 27% [9].

Possible explanations for the low prevalence in our study could be due to the good general health status [14] of the population that we examined, as determined in a previous study [11].

In our study,* Microsporum canis* was the more frequent isolated dermatophyte (86.7%) and this is in agreement with other feline studies conducted in Italy and worldwide [2, 5–7, 10, 15, 16].

We found no significant association between dermatophyte presence and sex, age, hair coat length, season of sampling, geographical habitat (i.e., rural or urban), or the presence or absence of parasites. Our results differ from the higher prevalence of young and long-haired cats found in previous studies [6, 17, 18]. The low overall prevalence of dermatophytes found in our study and the presence of only 5.1% long-haired cats in our sample might represent bias. Statistical analysis could be limited in some groups because of the sample size, and so some associations may be affected by type I errors; that is, in our case, no association was recorded between presence of the cats with dermatophytosis and age or no association was recorded between dermatophyte presence and cats hair coat length.

There were no positive cultures among the cats identified as positive (20/273) through examination using a Wood's lamp. It must be considered that the Wood's lamp facilitates identification of only 50% of the strains of* M. canis*, and this limitation may be particularly significant with the low positive prevalence found in our study [14, 16]. It must be emphasized that the field situation in which the research was done may have affected the accuracy of execution of the examination conducted by the Wood's lamp. In fact the environment was not completely dark and the analysis time was often reduced because the cats were anaesthetized.

No conclusions were possible with respect to an association between prevalence and breed because all the cats examined in this study were domestic shorthair (DSH). The overall number of cats with ectoparasites was low, and no relationship was found between the presence of ectoparasites and dermatophytosis prevalence, even though microtrauma resulting from pruritus induced by ectoparasites might predispose cats to* M. canis* infection [19, 20].

## 5. Conclusions

In conclusion, estimated prevalence of dermatophytes (*M. canis* and* T. mentagrophytes*) in stray cats of northern Italy was much lower than other Italian studies on the same population.

## Figures and Tables

**Table 1:** Epidemiological data of the population, prevalence of dermatophytes in relation to the epidemiological data and *P* value of Fisher's exact test and Odds ratio (OD) for each risk factor considered in a 273 stray cats with and without clinical signs living in north Italy.

Variable	Categories	Number of subject	%	Number (*N*) and percentage (%) of cats positive for dermatophytes	*P* value of Chi square test and Odds ratio (OD)
Sex	Female	199	72.9	14/199 (7.0%)	*P* = 0.067, OD = 5.52 (CI = 0.71–42.78)
	Male	74	27.1	1/74 (1.4%)	

Age	Young	120	44.0	9/120 (67.5%)	*P* = 0.198, OD = 1.99 (CI = 0.69–5.75)
	Adult	153	56.0	6/153 (3.9%)	

Breed	European domestic cats	273	100	15/273 (5.5%)	—

Habitat	Urban	149	54.6	11/149 (7.4%)	*P* = 0.133, OD = 2.39 (CI = 0.74–7.71)
	Rural	124	45.5	4/124 (3.2%)	

Coat	Short	259	94.9	14/259 (5.4%)	*P* = 0.781, OD =0.74 (CI = 0.09–6.09)
	Long	14	5.1	1/14 (7.1%)	

Season	Warm	129	47.3	4/129 (3.1%)	*P* = 0.100, OD = 0.39 (CI = 0.12–1.25)
	Cold	144	52.7	11/144 (7.6%)	

Skin lesion	Yes	63	23.1	3/63 (4.8%)	*P* = 0.770, OD = 0.83 (CI = 0.23–3.02)
	No	210	76.9	12/210 (5.7%)	

Positivity to dermatophytes	Yes	15	5.5	—	—
	No	258	94.5	—	—

Positivity to saprophytic fungi	Yes	201	73.6	2/201 (1.0%)	*P* = 0.0001*, OD = 0.05 (CI = 0.01–0.21)
	No	72	26.4	13/72 (18.1%)	

*P value < 0.05.
